# WP1130 attenuates cisplatin resistance by decreasing P53 expression in non–small cell lung carcinomas

**DOI:** 10.18632/oncotarget.16931

**Published:** 2017-04-07

**Authors:** Xiang Wang, Ying Bao, Zhaohui Dong, Qiuqiang Chen, Huihui Guo, Charlie Ziang, Jianzhong Shao

**Affiliations:** ^1^ College of Life Sciences, Zhejiang University, Hangzhou 310058, People's Republic of China; ^2^ Key Laboratory for Translational Medicine, First Affiliated Hospital, Huzhou University, Huzhou 313000, People's Republic of China; ^3^ State Key Laboratory for Diagnosis and Treatment of Infectious Diseases, and Collaborative Innovation Center for Diagnosis and Treatment of Infectious Diseases, The First Affiliated Hospital, School of Medicine, Zhejiang University, Hangzhou 310058, People's Republic of China

**Keywords:** non-small cell lung carcinomas, WP1130, cisplatin, p53, USP9X

## Abstract

Cisplatin-based combination chemotherapy significantly improves the survival outcomes in non–small cell lung carcinomas (NSCLCs), but drug resistance commonly contributes to disease progression and relapse. Recently, accumulating evidence has indicated that deubiquitinases (DUBs) are involved in regulating tumor cell proliferation, apoptosis, and chemoresistance. We designed this study to investigate the role of WP1130, a DUB inhibitor, in regulating cisplatin cytotoxicity in NSCLCs. After being combined with WP1130, cisplatin sensitivity was significantly increased in A549 and HCC827 cells with decreased p53 expression, inhibiting their proliferation, but not in p53-deficient NCI-H1299 cells. The synergistic cytotoxicity of the cisplatin and WP1130 co-treatment was abolished in p53-knockdown cells. Western blotting verified the decreased p53 expression in A549 and HCC827 cells treated with cisplatin and WP1130. The administration of MG132, a proteasome inhibitor, or knockdown of ubiquitin-specific peptidase 9, X-linked (USP9X) both eliminated the effect of WP1130 in decreasing p53 expression. Taken together, our findings confirm that the inclusion of WP1130 is potentially contributes to better therapeutic effects of cisplatin-based chemotherapy of NSCLCs in a manner dependent on the USP9X–p53 ubiquitination–mediated degradation pathway.

## INTRODUCTION

Lung cancer is the most prevalent cancer and the leading cause of cancer-related death worldwide [1]. According to the National Central Cancer Registry of China, incidence and mortality attributable to lung cancer accounted for 73.33% of all new cancer cases and 61.02% of all cancer-related mortality, respectively [2]. Based on its histological characteristics, lung cancer is divided into small cell lung carcinomas (SCLCs) and non–small cell lung carcinomas (NSCLCs); the latter accounts for an estimated 85% of lung cancer cases [3] and commonly presents with progression. Despite great advances in diagnosis and therapy, the long-term survival outcomes of lung cancer, especially with metastasis, remain poor. Notably, molecular targeted therapies have demonstrated a positive effect in improving progression-free survival outcomes in patients with advanced NSCLC [4, 5], but the incidence of epidermal growth factor receptor (*EGFR*) mutation and the *EML4*-*ALK* fusion gene is approximately 10% and only 4%, respectively [6, 7]. Therefore, only a small proportion of patients with NSCLCs would benefit from molecular targeted therapies, and patients who do not present drug-targetable driver mutations mostly receive platinum-based chemotherapy.

Compared with other platinum agents, cisplatin has greater activity and is used in chemotherapy for various cancers. Cisplatin was used for metastatic cutaneous squamous cell carcinoma and yielded an overall response rate of 45% and prolonged disease-free survival [8]. Cisplatin was also suitable for treating non-nasopharyngeal carcinoma, and every three weekly chemotherapy strategy yielded better 5-year overall survival outcomes as compared to a weekly chemotherapy strategy [9]. Cisplatin is used for most patients with NSCLCs and forms the basis of first-line chemotherapy [10]. Following cisplatin therapy, the 1- and 2-year disease-free survival rates of patients who underwent surgery for stage II–III NSCLCs were > 70% and 50%, respectively [11].

Although NSCLCs commonly have high chemosensitivity, the unavoidable disease recurrence suggests cisplatin resistance. Many efforts have been made to explore the mechanisms underlying lung cancer resistance to cisplatin treatment. Overexpressed PDA/PD-L1 not only decreased immunotherapy efficiency, but also increased lung cancer resistance to cisplatin [12]. In human ovarian cancer, upregulated zeste homolog 2 (EZH2) resulted in cisplatin by promoting cyclin-dependent kinases (CDK1, CDK2) and H3K27me3 [13]. In bladder cancer cells, the long non-coding RNA UCA1 increased cisplatin resistance by promoting microRNA (miR)-196a-5p expression targeting p27^kip1^ [14]. However, there remains the lack of an effective approach for overcoming NSCLC resistance to cisplatin.

Recently, several pieces of evidence have demonstrated that deubiquitinases (DUBs) are important for regulating cell proliferation, apoptosis, and chemoresistance. Ubiquitin-specific peptidase 9, X-linked (USP9X), a DUB family member, contributes to chemoresistance and disease relapse by stabilizing BCL2 family apoptosis regulator (MCL1) [15]. In aggressive B cell lymphoma, USP9X decreased the degradation of X-linked inhibitor of apoptosis protein (XIAP) to confer resistance against spindle poison–containing chemotherapy [16]. WP1130, a selective USP9X inhibitor, promotes apoptosis and has been considered as a potential chemosensitizer for combination chemotherapy [17]. Based on these findings, we designed this study to investigate whether WP1130 could attenuate cisplatin resistance and even decrease the cisplatin dosage for treating NSCLCs.

## RESULTS

### Effect of WP1130 co-treatment on cisplatin sensitivity in NSCLC cells

**Figure 1:**
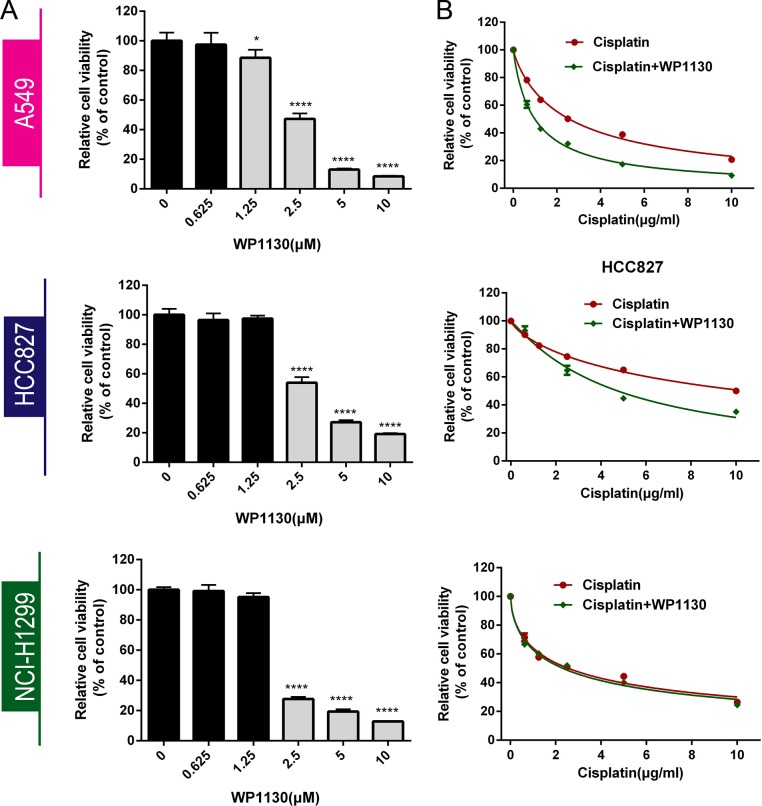
(**A**) CCK-8 was used to determine NSCLC cell viability following treatment with 0, 0.625, 1.25, 2.5, 5, or 10 μM WP1130 alone. WP1130 concentrations of ≥ 2.5 μM had significant inhibitory effects on NSCLC cell proliferation, but 0–1.25 μM WP1130 did not. *P < 0.05, ****P < 0.0001 vs. 0 μM WP1130. (**B**) WP1130 co-treatment increased A549 and HCC827 cell sensitivity to cisplatin. Cisplatin sensitivity in NCI-H1299 cells treated with cisplatin alone and with cisplatin and WP1130 co-treatment did not differ.

Three NSCLC cell lines (A549, HCC827, NCI-H1299) were incubated with 0–10 μM WP1130 to determine the IC50 by CCK-8 assay for 24 h, 48 h or 72 h (Supplementary Figure 1A–1B, Figure 1A). We chosen 48 h as the incubation time for all the next experiment. After 48 h incubation, WP1130 inhibited NSCLC cell proliferation significantly when the concentration are more than 2.5 μM, but 0–1.25 μM WP1130 did not (Figure 1A). The WP1130 IC50 in the A549, HCC827, NCI-H1299 cells was 2.5 μM, 2.5 μM, and 2.0 μM, respectively.

Following 48 h incubation with cisplatin in combination with WP1130, A549 and HCC827 cells presented enhanced cisplatin sensitivity. Cell viability was significantly decreased in the co-treatment group, and the cisplatin IC50 in the A549 and HCC827 cells was 2.5 μM and 10 μM, respectively, higher than the IC50 of 1 μM and 5 μM in the A549 and HCC827 cells that had been treated with WP1130. NCI-H1299 cells were more sensitive to WP1130 than the A549 and HCC827 cells, but did not benefit from the cisplatin and WP1130 co-treatment (Figure 1B).

### WP1130 improved cisplatin resistance by decreasing p53 expression

**Figure 2:**
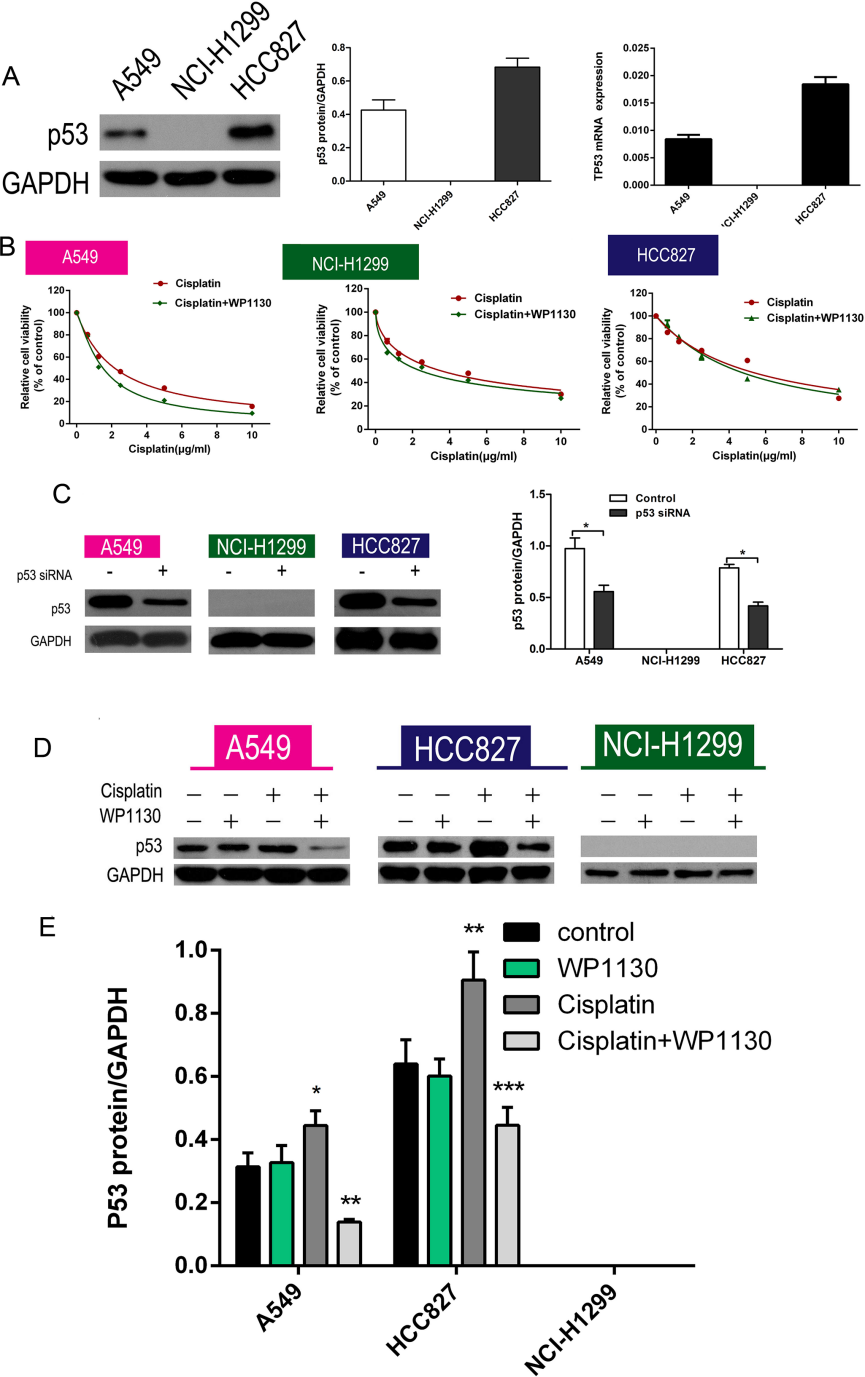
(**A**) Western blotting and RT-PCR verified that p53 expression was highest in HCC827 cells, moderate in A549 cells, and absent in NCI-H1299 cells. (**B**) No significant difference between cisplatin alone and cisplatin and WP1130 co-treatment in NCI-H1299, A549, and HCC827 cells with p53 knockdown. (**C**) Western blotting confirmed the efficiency of siRNA knockdown of p53. *P < 0.05 vs. Control. (**D**, **E**) Western blotting was used to detect the expression of p53 in NSCLC cells. Cisplatin increased p53 expression in A549 and HCC827 cells; WP1130 co-treatment reversed the effect, but WP1130 alone did not inhibit p53 expression in the NSCLC cell lines. *P < 0.05, **P < 0.01, ***P < 0.001 vs. Control.

Of the three NSCLC cell lines, only NCI-H1299 cells did not express p53; RT-PCR and western blotting were used to detect p53 expression in the NSCLC cells and confirmed no p53 expression in the NCI-H1299 cells (Figure 2A). Furthermore, p53 expression was markedly upregulated in A549 and HCC827 cells after cisplatin administration. To further confirm that p53 is a key regulator of WP1130 promotion of cisplatin sensitivity, the NSCLC cells were treated with cisplatin alone or cisplatin plus WP1130 after p53 knockdown. The results suggested that p53 knockdown eliminated the effect of WP1130 in increasing cisplatin sensitivity (Figure 2B). Western blotting confirmed the efficiency of short interfering RNA (siRNA) knockdown of p53 (Figure 2C). Furthermore, WP1130 co-treatment reversed the effect of cisplatin on increasing p53 expression, but WP1130 alone did not inhibit p53 expression in the NSCLC cells (Figure 2D, 2E and Supplementary Figure 2C) and *in vivo* (Supplementary Figure 2A). Furthermore, we found that WP1130 have no effect on the expression of MDM2 in the NSCLC cells (Supplementary Figure 2D).

### The role of WP1130 in NSCLC cell proliferation and apoptosis

**Figure 3:**
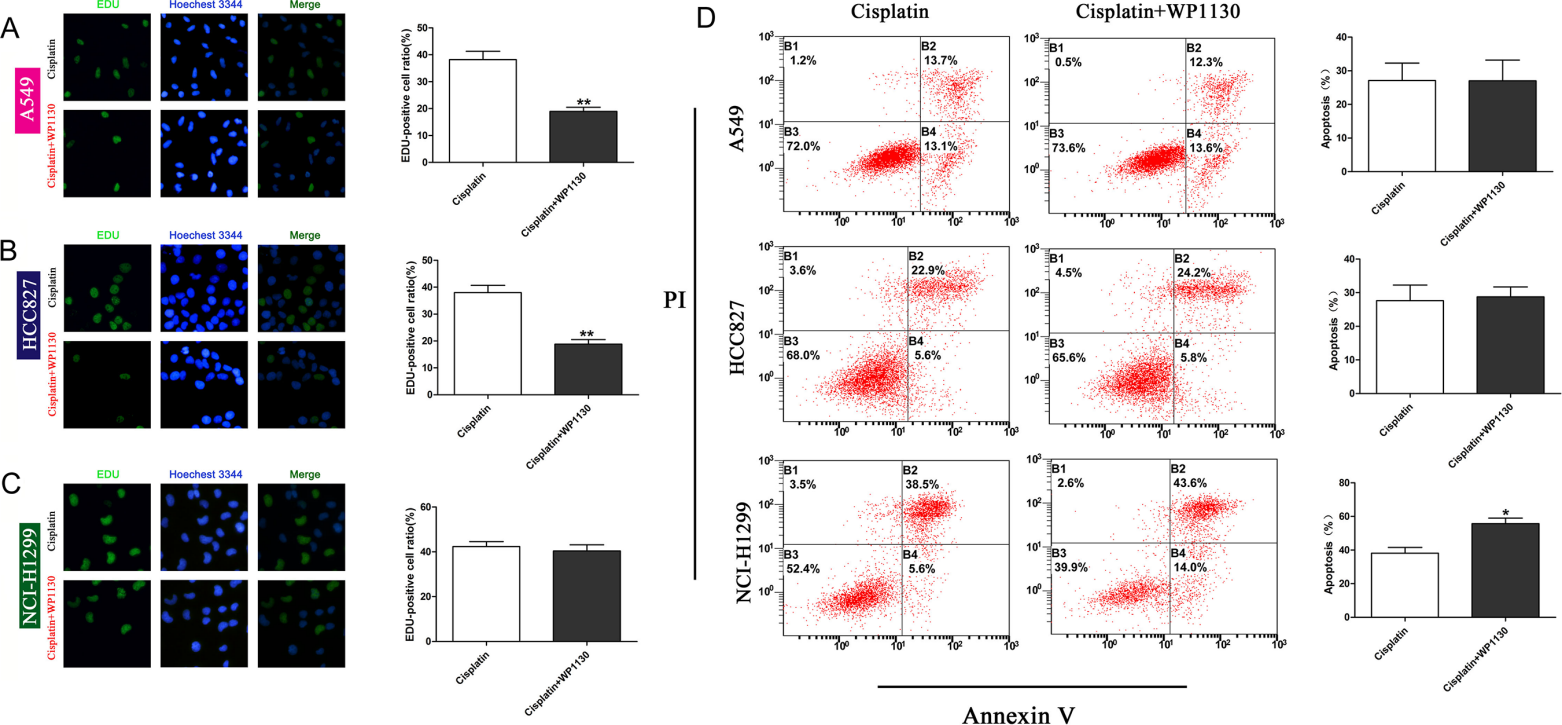
Images and quantification of EdU staining following 48-h treatment with cisplatin and cisplatin plus WP1130 Compared with cisplatin alone, WP1130 and cisplatin co-treatment reduced the proliferative ability of A549 cells (**A**) and HCC827 cells (**B**) in an obvious manner, but not that of NCI-H1299 cells (**C**). **P < 0.01. (**D**) Apoptotic NSCLC cells were determined with flow cytometry following treatment with cisplatin alone or cisplatin combined with WP1130. WP1130 and cisplatin co-treatment increased the apoptosis rate of NCI-H1299 cells, but not that of A549 and HCC827 cells. *P < 0.05 vs. cisplatin.

No matter DUBs or p53 is involved in regulating cell proliferation and apoptosis. We used the EdU incorporation assay and flow cytometry to determine the effect of WP1130 on NSCLC cell proliferation and apoptosis, respectively. Compared with the controls, the proliferation ability of the A549 and HCC827 cells was obviously reduced following WP1130 and cisplatin co-treatment, but that of the NCI-H1299 cells was not (Figure 3A–3C). In contrast to the proliferation experiment, WP1130 and cisplatin co-treatment did not increase the apoptosis rate of the A549 and HCC827 cells, but apparently promoted NCI-H1299 cell apoptosis (Figure 3D). These results suggest that the WP1130 downregulation of p53 expression sensitizes NSCLC cells to cisplatin mainly by inhibiting cell proliferation.

### WP1130 reduced NSCLC cell resistance to cisplatin in a USP9X-dependent manner

**Figure 4:**
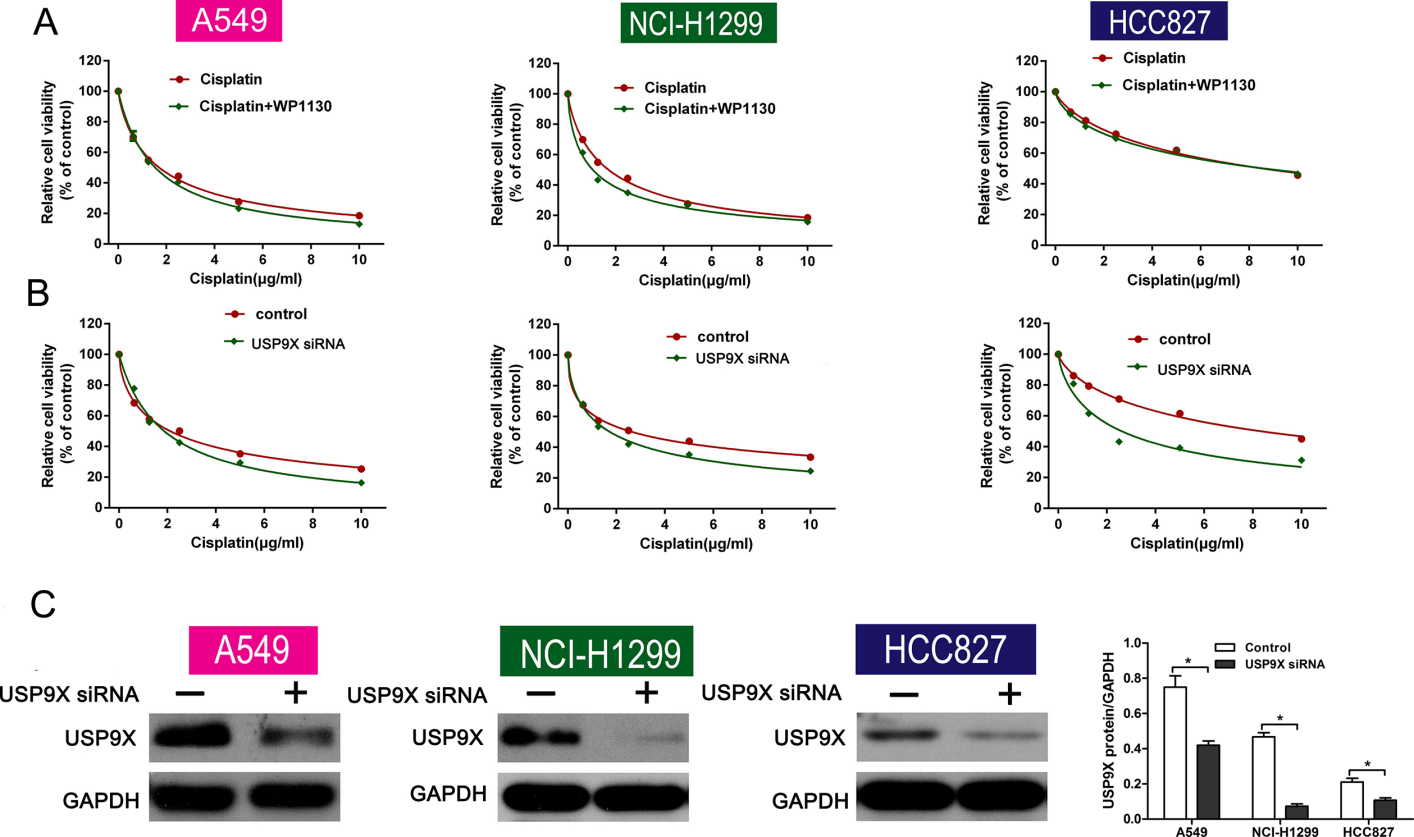
(**A**) Cell viability between the cisplatin-alone and co-treatment groups was not different following USP9X knockdown. NSCLC cell viability was determined using CCK-8. (**B**) USP9X knockdown led to increased inhibition of cell viability after cisplatin administration. CCK-8 was used to determine NSCLC cell viability. (**C**) Western blotting verified the efficiency of USP9X knockdown. *P < 0.05 vs. Control.

USP9X correlates with chemoresistance and is a selective target of WP1130. To determine whether the deubiquitination activities of USP9X are necessary for the effect of WP1130, USP9X was knocked down with siRNA in NSCLC cells. Consequently, there was no significant change in cell viability between the cisplatin-only group and the co-treatment group (Figure 4A). Furthermore, USP9X knockdown increased the inhibition of cell viability after cisplatin administration (Figure 4B). Western blotting verified the efficiency of USP9X knockdown (Figure 4C). And the expression of p53 was not altered after transfecting with USP9X siRNA (Supplementary Figure 2B) as WP1130 treatment).

### USP9X inhibited p53 ubiquitination–mediated degradation

**Figure 5:**
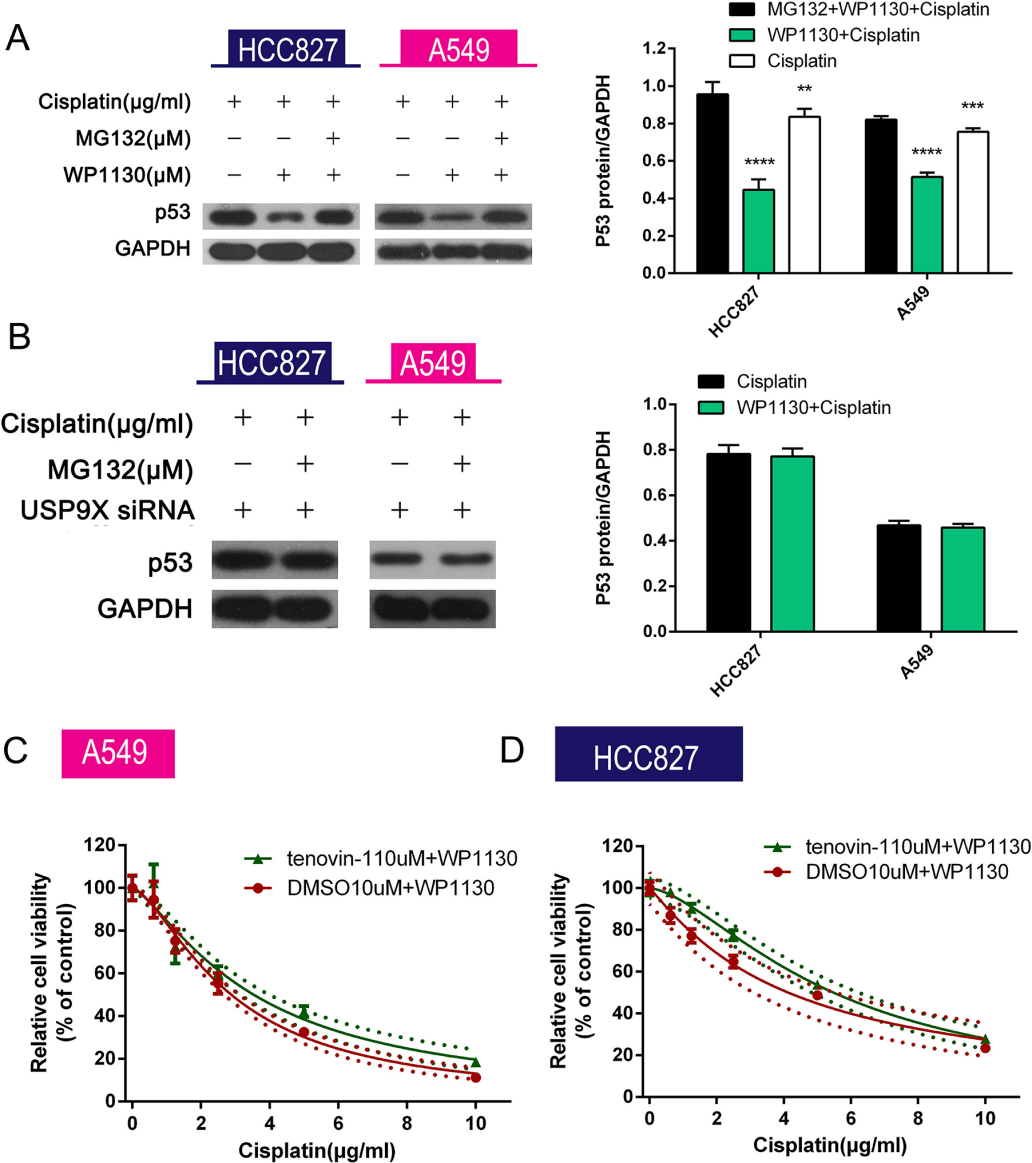
(**A**) MG132 reversed the decreased p53 expression in A549 and HCC827 cells co-treated with cisplatin and WP1130. Western blotting was performed to detect p53 expression in NSCLC cells. *P < 0.05 vs. cisplatin, ^#^*P* < 0.05 vs. cisplatin+WP1130. (**B**) USP9X siRNA–transfected NSCLC cells were exposed to cisplatin alone or in combination with MG132 for 48 h followed by measurement of p53 expression in the cells. USP9X knockdown abolished the inhibitory effect of WP1130 on p53 expression in A549 and HCC827 cells treated with cisplatin plus MG132. (**C**, **D**) WP1130 and cisplatin were co-administered to NSCLC cell lines pretreated or untreated with tenovin-1. Cell viability was measured by CCK-8. Tenovin-1 increased the cisplatin resistance of A549 and HCC827 cells in the presence of WP1130.

MG132, a proteasome/calpain inhibitor, was added to the A549 and HCC827 cell culture medium that had been supplemented with WP1130 and cisplatin. Compared with the co-treatment group, p53 expression was upregulated in the triple combination (cisplatin plus WP1130 plus MG132) group (Figure 5A). USP9X knockdown eliminated the decreased p53 expression caused by WP1130 in the presence of cisplatin (Figure 5B). These results imply that WP1130 increases p53 degradation through the ubiquitin proteasome pathway and is dependent on USP9X. To further verify this, we administered tenovin-1, a MDM2 inhibitor that inhibits p53 ubiquitination–mediated degradation, and it significantly increased the cisplatin resistance of the WP1130-treated A549 and HCC827 cells (Figure 5C).

### WP1130 increased the inhibitory effect of cisplatin on tumorigenesis *in vivo*

**Figure 6:**
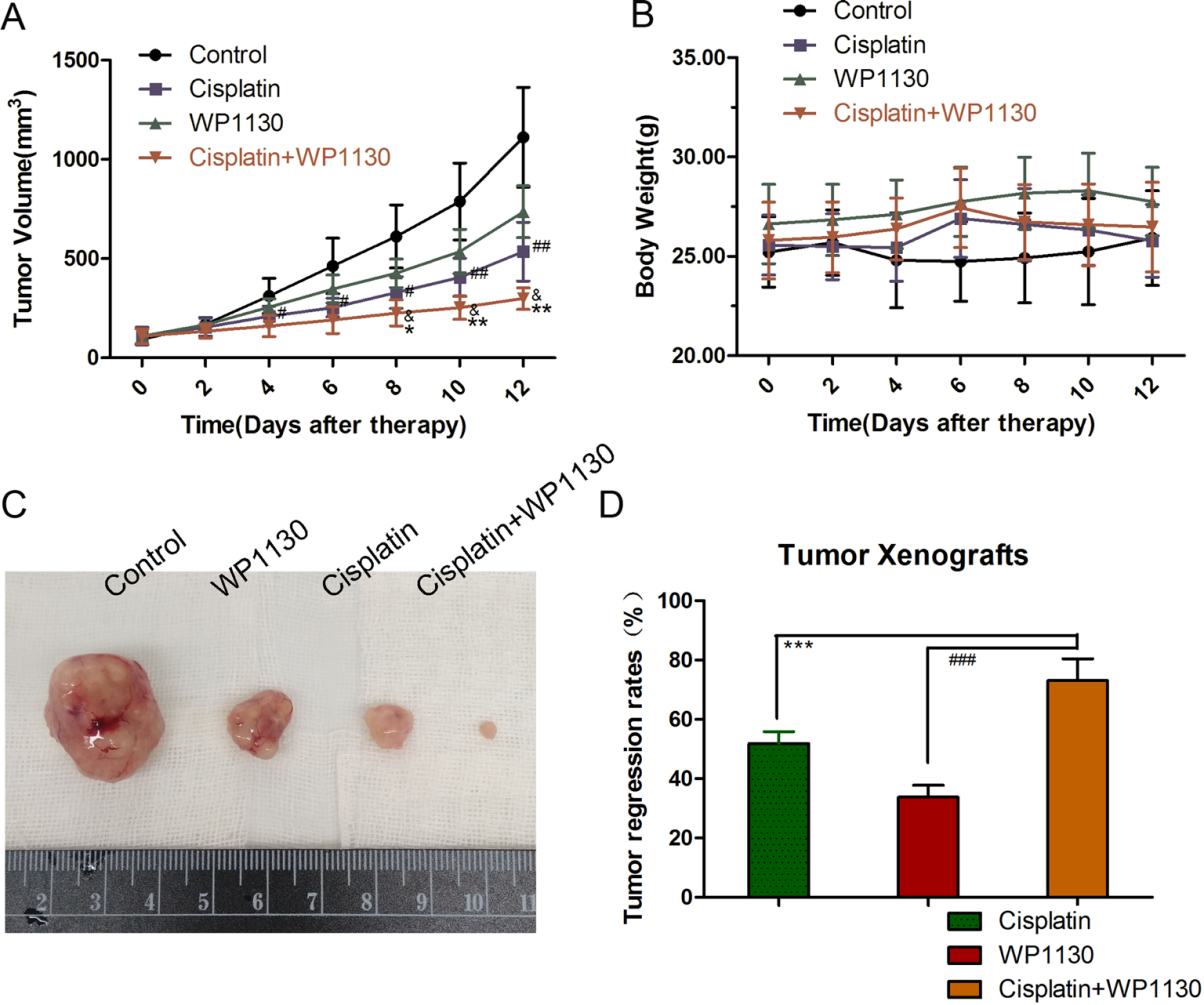
(**A**) Tumor volumes ranked from large to small: blank group, WP1130 group, cisplatin group, and co-treatment group. (**B**) The body weights of the mice were not significantly different following treatment. (**C**) Compared with the control, treatment with cisplatin or WP1130 alone delayed tumor growth and decreased tumor size and weight. (**D**) Tumor regression rate in the co-treatment group was significantly higher compared to that in the single-reagent groups.

To characterize the cisplatin and WP1130 co-treatment *in vivo*, A549 cells were xenografted in to immunodeficient mice. The body weights of the mice were not significantly changed at the end of the treatment (Figure 6B). Compared with the control, treatment with cisplatin or WP1130 alone had inhibitory effects on tumorigenesis *in vivo*, where, similarly to the *in vitro* studies, there was delayed tumor growth and decreased tumor size and weight (Figure 6A, 6C). The tumor regression rate of the co-treatment group was significantly higher than that of the single-treatment groups, which implies that cisplatin and WP1130 co-administration has a synergistic anti-tumor effect (Figure 6D). Together, these results suggest that WP1130 increases cisplatin sensitivity *in vivo*.

## DISCUSSION

A concerted effort has been made to refine the use of cisplatin in the chemotherapy of NSCLCs over the years. Unlike molecular targeted therapies that have small proportions of candidates, cisplatin-based chemotherapy is administered to almost all patients with advanced NSCLCs. A meta-analysis revealed that cisplatin reduces the risk of NSCLC-related death by 27% and increases the rate of 1-year survival by 10% [18]. Furthermore, a clinical trial confirmed that cisplatin-based combination chemotherapy significantly improves patient quality of life [19]. A series of randomized trials with large sample sizes also demonstrated that cisplatin-based chemotherapy is associated with improved survival outcomes in NSCLCs [20–22]. These studies recommended cisplatin-based doublets as standard care. However, the increasing cisplatin resistance limits the survival benefits and promotes tumor progression and relapse. Improving cisplatin sensitivity is necessary for prolonging the duration of disease-free survival and for reducing the cisplatin dosage; the latter is also helpful for increasing patients' tolerance by avoiding serious adverse drug reactions such as nephrotoxicity, emesis, and renal impairment.

Recently, it was revealed that DUBs affect the resistance of many cancer cell types to spindle poisons or doxorubicin by inhibiting ubiquitination-mediated degradation of key proteins [15, 16, 23]. WP1130, a DUB inhibitor, has an inhibitory effect on tumor growth by blocking autophagy and inducing apoptosis [17, 24], and it reduced chemotherapy resistance of hepatocellular carcinoma [23]. Therefore, DUBs are considered potential chemosensitizers or therapeutic targets. In the present study, WP1130 and cisplatin co-treatment had a synergistic inhibitory effect on A549 and HCC827 cell viability, but not NCI-H1299 cell viability. The p53 expression levels between the NCI-H1299 cells and the A549 and HCC827 cells differed greatly. After cisplatin treatment, p53 expression was markedly upregulated in the A549 and HCC827 cells but was inhibited following cisplatin and WP1130 co-treatment. These results suggest that p53 is necessary to the WP1130-increased cisplatin sensitivity in NSCLC cells.

Several studies have demonstrated that DUBs regulate intracellular p53 protein levels. Abraxas brother 1 (ABRO1), a component of the BRCC36-containing isopeptidase complex (BRISC), stabilizes p53 by facilitating the interaction of p53 with USP7 to reduce p53 ubiquitination [25]. Moreover, USP15 knockdown delayed p53 ubiquitination–mediated degradation in an obvious manner by accelerating MDM2 degradation in A375 cells [26]. Based on WP1130 selective inhibition of DUBs, including USP9X, USP5, USP14, and UCH37, we investigated whether WP1130-increased chemosensitivity was dependent on USP9X. Our results show that USP9X knockdown eliminated the synergistic effect of WP1130 plus cisplatin in NSCLC cells. However, USP9X knockdown increased NSCLC cell sensitivity to cisplatin, as it did with WP1130. Together, these results imply that WP1130 decreases cisplatin resistance by inhibiting the USP9X–p53 ubiquitination pathways.

Ubiquitination or deubiquitination closely regulate intracellular p53 protein levels [27]. We used the proteasome inhibitor MG132 to further verify the p53 downregulation through proteasome-dependent degradation after p53 ubiquitination. Western blotting revealed that MG132 reversed the inhibitory effect of WP1130 on p53 expression in the NSCLC cells following cisplatin treatment. Tenovin-1, an inhibitor of MDM2 (the most studied promoter of p53 ubiquitination), protects p53 from ubiquitination-mediated degradation, and only in cells with wild-type p53 status. In the present study, tenovin-1 (a wild-type p53 activator) pretreatment decreased NSCLC cell sensitivity to cisplatin and WP1130 co-treatment, indicating that the USP9X–p53 ubiquitination/degradation pathway is necessary for WP1130-mediated chemosensitization.

The tumor suppressor p53 is a transcriptional factor that promotes apoptosis, cell cycle arrest, and DNA repair in response to cellular stress. Information on the correlation between p53 and DNA-crosslinking agents is conflicting because p53 potentially decreases the effect of DNA poisons, leading to drug resistance [28]. For example, upregulated p53 increased doxorubicin resistance of hepatocellular carcinoma [23]. Nevertheless, several pieces of evidence support the premise that increased p53 expression optimizes cisplatin treatment efficacy by inducing apoptosis in various cancer cell types [29, 30]. Several mechanisms are response to the contradictory role of p53 in regulating cisplatin sensitivity. p53 overexpression increases chemotherapy sensitivity by increasing the expression and activation of apoptosis-related proteins such as caspase-3, caspase-7, and Bax. However, cycle arrest and DNA repair are increased in tumor cells resistant to DNA poisons. In the present study, p53 downregulation did not decrease the apoptosis rate following WP1130 and cisplatin co-treatment. This result suggests that inhibiting DUBs leads to p53 tending toward cell cycle arrest and DNA repair rather than apoptosis.

Moreover, the presence of mutated p53 rendered the NSCLC cells more resistant to cisplatin and increased the cisplatin IC50 [31]. Mutation within the *P53* gene is one of the most common genetic alterations in more than 50% of NSCLCs [32]. Therefore, combining WP1130 with decreased expression of mutated p53 would further increase the therapeutic effect of cisplatin in clinical practice.

In conclusion, the present study confirms that WP1130 co-treatment increases cisplatin cytotoxicity by stabilizing p53 and reducing p53 ubiquitination–mediated degradation in a USP9X-dependent manner. The combination with WP1130 potentially contributes to a better therapeutic effect of cisplatin-based chemotherapy for patients with NSCLCs.

## MATERIALS AND METHODS

### Cell culture

Three human NSCLC cell lines (A549, HCC827, NCI-H1299) were obtained from the Shanghai Institutes of Biological Sciences (Shanghai, China) and cultured according to the instructions provided. All cells were incubated in RPMI 1640 complete medium (Gibco-Invitrogen, Carlsbad, CA, USA) containing 10% fetal bovine serum (FBS), penicillin (100 U/mL), and streptomycin (100 mg/mL) in 5% CO_2_ at 37°C.

### Cell viability assay

The cells (3 × 10^3^/well) were seeded in 96-well plates. After 24-h starvation in serum-free medium, RPMI 1640 medium containing 10% FBS and drugs (cisplatin and WP1130) were used for subsequent 48-h incubation. Then, fresh complete medium containing Cell Counting Kit-8 (CCK-8) solution (10:1, Dojindo, Kumamoto, Japan) was added to the plates for 3-h incubation. Cell viability was determined by the absorbance at 450 nm with an MRX II microplate reader (Dynex Technology, Chantilly, VA, USA).

### siRNA transfection

NSCLC cells were transfected with USP9X siRNA (100 nM) or p53 siRNA (100 nM; Santa Cruz Biotechnology, Dallas, TX, USA) using Lipofectamine 2000 (Invitrogen, Carlsbad, CA, USA) according to the manufacturer's protocol. The transfection medium was replaced with complete medium 6 h after transfection, and the cells were incubated for the indicated times. All treatments were started 24 h after transfection.

### Ethynyl deoxyuridine (EdU) incorporation assay

The cell proliferation rate was calculated using a Click-iT EdU Imaging Kit (Invitrogen, Carlsbad, CA, USA) according to the manufacturer's instructions. Briefly, cells were plated in 96-well plates at 4 × 10^5^ cells per well. EdU (50 μM, 100 μL/well) was added to the plates for 2-h incubation. After washing three times, the cells were fixed with 4% paraformaldehyde. Apollo^®^ fluorescent dye solution (Invitrogen, Carlsbad, CA, USA) was added and the plates were incubated for 30 min, and cell proliferation was observed under fluorescence microscopy.

### Apoptosis assay

Cells were exposed to cisplatin [median inhibitory concentration, IC50 (μg/ml) A549 cells, 2.5; HCC827 cells, 10; NCI-H1299 cells, 2.4] alone or to cisplatin and WP1130 (A549 cells, 2.5 μM; HCC827 cells, 2.5 μM; NCI-H1299 cells, 2.0 μM). After 48-h incubation, the cells were centrifuged and collected. After washing twice with phosphate-buffered saline (PBS), the cells were resuspended in 100 μL PBS and incubated with an Apoptosis Detection Kit (BD, Franklin Lakes, NJ, USA) according to the manufacturer's instructions. The apoptosis rate was calculated using flow cytometry.

### Quantitative real-time reverse transcription–PCR (RT-PCR)

Total RNA was isolated using TRIzol (Invitrogen) according to the manufacturer's protocol, and subsequent reverse transcription was performed using a ReverTra Ace -α- reverse transcription kit (Invitrogen). Real-time PCR was performed according to the SYBR Premix Ex Taq kit (Takara, Shiga, Japan) protocol in a Roche LightCycler system (Roche, Basel, Switzerland). The primers utilized for the real-time PCR are as follows: p53: Forward 5′-TCA GCATCTTATCCGAGTGGAA-3′ Reverse 5′-TGTAGT GGATGGTGGTACAGTCA-3′ USP9X: Forward 5′-CAAT GGATAGATCGCTTTATA-3′ Reverse 5′-CTTCTTG CCATGGCCTTAAAT-3′ Glyceraldehyde-3-phosphate dehydrogenase (GAPDH): Forward 5′-CGGAGTCAA CGGATTTGGTCGTAT-3′ Reverse 5′-AGCCTTCTCCA TGGTGGTGAAGAC-3′

### Western blotting

Cells were lysed in radioimmunoprecipitation assay buffer containing protease inhibitors and were quantified using a bicinchoninic acid kit (Thermo Fisher Scientific, Rockford, IL, USA). Equal amounts of protein were separated by sodium dodecyl sulfate–polyacrylamide gel electrophoresis and then transferred to 0.45-μm polyvinylidene fluoride membranes (Millipore, Bedford, MA, USA). The membranes were blocked using Tris-buffered saline (TBS) and 0.1% Tween 20 (TBST) containing 5% bovine serum albumin for 2 h and then incubated with primary antibodies (anti-p53, -USP9X, and -GAPDH, diluted 1:1000 in TBST) overnight. After washing three times with TBST, the membranes were incubated with the appropriate secondary antibodies conjugated to horseradish peroxidase for 1 h at room temperature. Bands were visualized using enhanced chemiluminescence in the western blot detection system. All antibodies were purchased from Abcam (Cambridge, MA, USA).

### Tumor xenograft experiments

The experimental procedures were conducted in conformity with institutional guidelines for the care and use of laboratory animals of the First Affiliated Hospital of Hu Zhou University, Huzhou, China, and conformed to the National Institutes of Health Guide for Care and Use of Laboratory Animals (NIH Publications, No. 8023, revised 1978). A mouse model of NSCLC was established using A549 cells to generate subcutaneous xenografts. Briefly, approximately 5 × 10^6^ cells per mouse were injected subcutaneously into the lateral flanks of immunodeficient mice. There were six mice in each group. The tumor volume was measured and calculated as follows: volume = (width^2^ × length)/2. The drugs were administered to the mice when the tumor volumes were about 100 mm^3^. Mice were randomly divided into three groups: intraperitoneally injected with 2 mg/kg cisplatin on alternate days, intraperitoneally injected with 20 mg/kg WP1130 twice weekly, or co-treated with cisplatin with WP1130. After 6 weeks, the mice were sacrificed and the tumor xenografts were harvested.

### Statistical analysis

All data are presented as the mean ± SD and frequency. The difference between groups was analyzed by two-tailed Student's *t*-test and Fisher's exact test. Statistical analysis was performed using SPSS19.0 software (SPSS Inc., Chicago, IL, USA). Differences were considered statistically significant at *p* < 0.05.

## SUPPLEMENTARY FIGURES



## References

[1] Torre LA, Bray F, Siegel RL, Ferlay J, Lortet-Tieulent J, Jemal A (2015). Global cancer statistics, 2012. CA Cancer J Clin.

[2] Chen W, Zheng R, Baade PD, Zhang S, Zeng H, Bray F, Jemal A, Yu XQ, He J (2016). Cancer statistics in China, 2015. CA Cancer J Clin.

[3] Parsons A, Daley A, Begh R, Aveyard P (2010). Influence of smoking cessation after diagnosis of early stage lung cancer on prognosis: systematic review of observational studies with meta-analysis. BMJ.

[4] Maemondo M, Inoue A, Kobayashi K, Sugawara S, Oizumi S, Isobe H, Gemma A, Harada M, Yoshizawa H, Kinoshita I, Fujita Y, Okinaga S, Hirano H (2010). Gefitinib or chemotherapy for non-small-cell lung cancer with mutated EGFR. N Engl J Med.

[5] Greenhalgh J, Dwan K, Boland A, Bates V, Vecchio F, Dundar Y, Jain P, Green JA (2016). First-line treatment of advanced epidermal growth factor receptor (EGFR) mutation positive non-squamous non-small cell lung cancer. Cochrane Database Syst Rev.

[6] Reck M, Popat S, Reinmuth N, De Ruysscher D, Kerr KM, Peters S, ESMO Guidelines Working Group (2014). Metastatic non-small-cell lung cancer (NSCLC): ESMO Clinical Practice Guidelines for diagnosis, treatment and follow-up. Ann Oncol.

[7] Shaw AT, Kim DW, Nakagawa K, Seto T, Crinó L, Ahn MJ, De Pas T, Besse B, Solomon BJ, Blackhall F, Wu YL, Thomas M, O'Byrne KJ (2013). Crizotinib versus chemotherapy in advanced ALK-positive lung cancer. N Engl J Med.

[8] Trodello C, Pepper JP, Wong M, Wysong A (2017). Cisplatin and Cetuximab Treatment for Metastatic Cutaneous Squamous Cell Carcinoma: A Systematic Review. Dermatol Surg.

[9] Guan J, Zhang Y, Li Q, Zhang Y, Li L, Chen M, Xiao N, Chen L (2016). A meta-analysis of weekly cisplatin versus three weekly cisplatin chemotherapy plus concurrent radiotherapy (CRT) for advanced head and neck cancer (HNC). Oncotarget.

[10] Fennell DA, Summers Y, Cadranel J, Benepal T, Christoph DC, Lal R, Das M, Maxwell F, Visseren-Grul C, Ferry D (2016). Cisplatin in the modern era: the backbone of first-line chemotherapy for non-small cell lung cancer. Cancer Treat Rev.

[11] Chen Q, Ji X, Zhou X, Shi Q, Yu H, Fu H (2015). Clinical Observation of Docetaxel or Gemcitabine Combined with Cisplatin in the Chemotherapy after Surgery for Stage II–III Non-small Cell Lung Cancer. W Indian Med J. West Indian Med J.

[12] Yan F, Pang J, Peng Y, Molina JR, Yang P, Liu S (2016). Elevated Cellular PD1/PD-L1 Expression Confers Acquired Resistance to Cisplatin in Small Cell Lung Cancer Cells. PLoS One.

[13] Sun Y, Jin L, Liu JH, Sui YX, Han LL, Shen XL (2016). Interfering EZH2 Expression Reverses the Cisplatin Resistance in Human Ovarian Cancer by Inhibiting Autophagy. Cancer Biother Radiopharm.

[14] Pan J, Li X, Wu W, Xue M, Hou H, Zhai W, Chen W (2016). Long non-coding RNA UCA1 promotes cisplatin/gemcitabine resistance through CREB modulating miR-196a-5p in bladder cancer cells. Cancer Lett.

[15] Schwickart M, Huang X, Lill JR, Liu J, Ferrando R, French DM, Maecker H, O'Rourke K, Bazan F, Eastham-Anderson J, Yue P, Dornan D, Huang DC, Dixit VM (2010). Deubiquitinase USP9X stabilizes MCL1 and promotes tumour cell survival. Nature.

[16] Engel K, Rudelius M, Slawska J, Jacobs L, Ahangarian Abhari B, Altmann B, Kurutz J, Rathakrishnan A, Fernández-Sáiz V, Brunner A, Targosz BS, Loewecke F, Gloeckner CJ (2016). USP9X stabilizes XIAP to regulate mitotic cell death and chemoresistance in aggressive B-cell lymphoma. EMBO Mol Med.

[17] Kapuria V, Peterson LF, Fang D, Bornmann WG, Talpaz M, Donato NJ (2010). Deubiquitinase inhibition by small-molecule WP1130 triggers aggresome formation and tumor cell apoptosis. Cancer Res.

[18] Non-small Cell Lung Cancer Collaborative Group (1995). Chemotherapy in non-small cell lung cancer: a meta-analysis using updated data on individual patients from 52 randomised clinical trials. BMJ.

[19] Cullen MH, Billingham LJ, Woodroffe CM, Chetiyawardana AD, Gower NH, Joshi R, Ferry DR, Rudd RM, Spiro SG, Cook JE, Trask C, Bessell E, Connolly CK (1999). Mitomycin, ifosfamide, and cisplatin in unresectable non-small-cell lung cancer: effects on survival and quality of life. J Clin Oncol.

[20] Thongprasert S, Sanguanmitra P, Juthapan W, Clinch J (1999). Relationship between quality of life and clinical outcomes in advanced non-small cell lung cancer: best supportive care (BSC) versus BSC plus chemotherapy. Lung Cancer.

[21] Helsing M, Bergman B, Thaning L, Hero U, Joint Lung Cancer Study Group (1998). Quality of life and survival in patients with advanced non-small cell lung cancer receiving supportive care plus chemotherapy with carboplatin and etoposide or supportive care only. A multicentre randomised phase III trial. Eur J Cancer.

[22] Schiller JH, Harrington D, Belani CP, Langer C, Sandler A, Krook J, Zhu J, Johnson DH, Eastern Cooperative Oncology Group (2002). Comparison of four chemotherapy regimens for advanced non-small-cell lung cancer. N Engl J Med.

[23] Liu H, Chen W, Liang C, Chen BW, Zhi X, Zhang S, Zheng X, Bai X, Liang T (2015). WP1130 increases doxorubicin sensitivity in hepatocellular carcinoma cells through usp9x-dependent p53 degradation. Cancer Lett.

[24] DrieΔen S, Berleth N, Friesen O, Löffler AS, Böhler P, Hieke N, Stuhldreier F, Peter C, Schink KO, Schultz SW, Stenmark H, Holland P, Simonsen A (2015). Deubiquitinase inhibition by WP1130 leads to ULK1 aggregation and blockade of autophagy. Autophagy.

[25] Zhang J, Cao M, Dong J, Li C, Xu W, Zhan Y, Wang X, Yu M, Ge C, Ge Z, Yang X (2014). ABRO1 suppresses tumourigenesis and regulates the DNA damage response by stabilizing p53. Nat Commun.

[26] Zou Q, Jin J, Hu H, Li HS, Romano S, Xiao Y, Nakaya M, Zhou X, Cheng X, Yang P, Lozano G, Zhu C, Watowich SS (2014). USP15 stabilizes MDM2 to mediate cancer-cell survival and inhibit antitumor T cell responses. Nat Immunol.

[27] Brooks CL, Gu W (2006). p53 ubiquitination: Mdm2 and beyond. Mol Cell.

[28] Viktorsson K, De Petris L, Lewensohn R (2005). The role of p53 in treatment responses of lung cancer. Biochem Biophys Res Commun.

[29] Leekha A, Gurjar BS, Tyagi A, Rizvi MA, Verma AK (2016). Vitamin C in synergism with cisplatin induces cell death in cervical cancer cells through altered redox cycling and p53 upregulation. J Cancer Res Clin.

[30] Perdomo JA, Naomoto Y, Haisa M, Fujiwara T, Hamada M, Yasuoka Y, Tanaka N (1998). In vivo influence of p53 status on proliferation and chemoradiosensitivity in non-small-cell lung cancer. J Cancer Res Clin Oncol.

[31] Lai SL, Perng RP, Hwang J (2000). p53 gene status modulates the chemosensitivity of non-small cell lung cancer cells. J Biomed Sci.

[32] Brambilla C, Fievet F, Jeanmart M, de Fraipont F, Lantuejoul S, Frappat V, Ferretti G, Brichon PY, Moro-Sibilot D (2003). Early detection of lung cancer: role of biomarkers. Eur Respir J Suppl.

