# Supplementary material for: WP1130 attenuates cisplatin resistance by decreasing P53 expression in non–small cell lung carcinomas
Source: Oncotarget. 2017 Apr 7;8(30):49033–43. doi: 10.18632/oncotarget.16931 (PMC5564746; doi:10.18632/oncotarget.16931)
Supplement: Supplementary file 1 [file oncotarget-08-49033-s001.pdf]

WP1130 attenuates cisplatin resistance by decreasing P53 expression in non–small cell lung carcinomas

Supplementary Materials

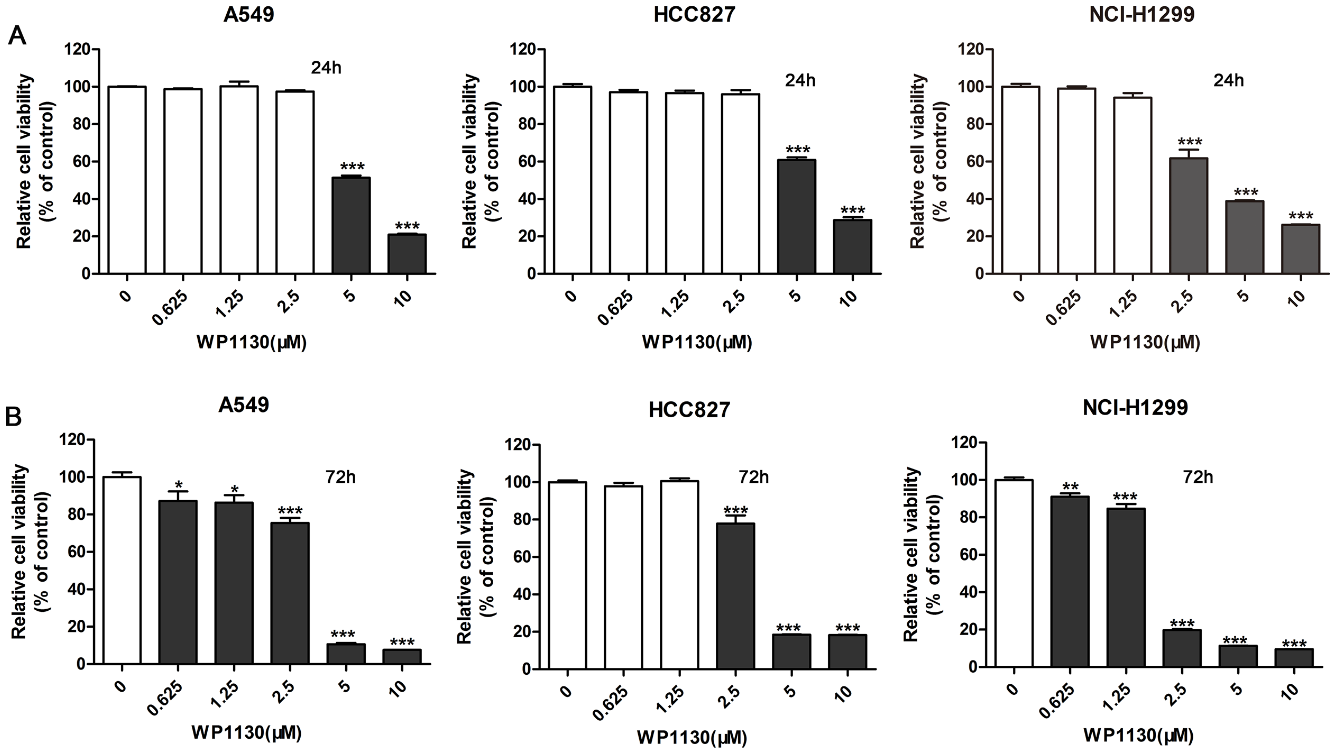

Supplementary Figure 1: (**A**)We used CCK-8 to determine NSCLC cell viability following treatment with 0, 0.625, 1.25, 2.5, 5, or 10 µM WP1130 alone for 24 h. ****P* < 0.0001 vs. 0 µM WP1130. (**B**) CCK-8 was used to examine NSCLC cell viability following treatment with 0, 0.625, 1.25, 2.5, 5, or 10 µM WP1130 alone for 72 h. **P* < 0.05, ***P* < 0.001 ,****P* < 0.0001 vs. 0 µM WP1130.

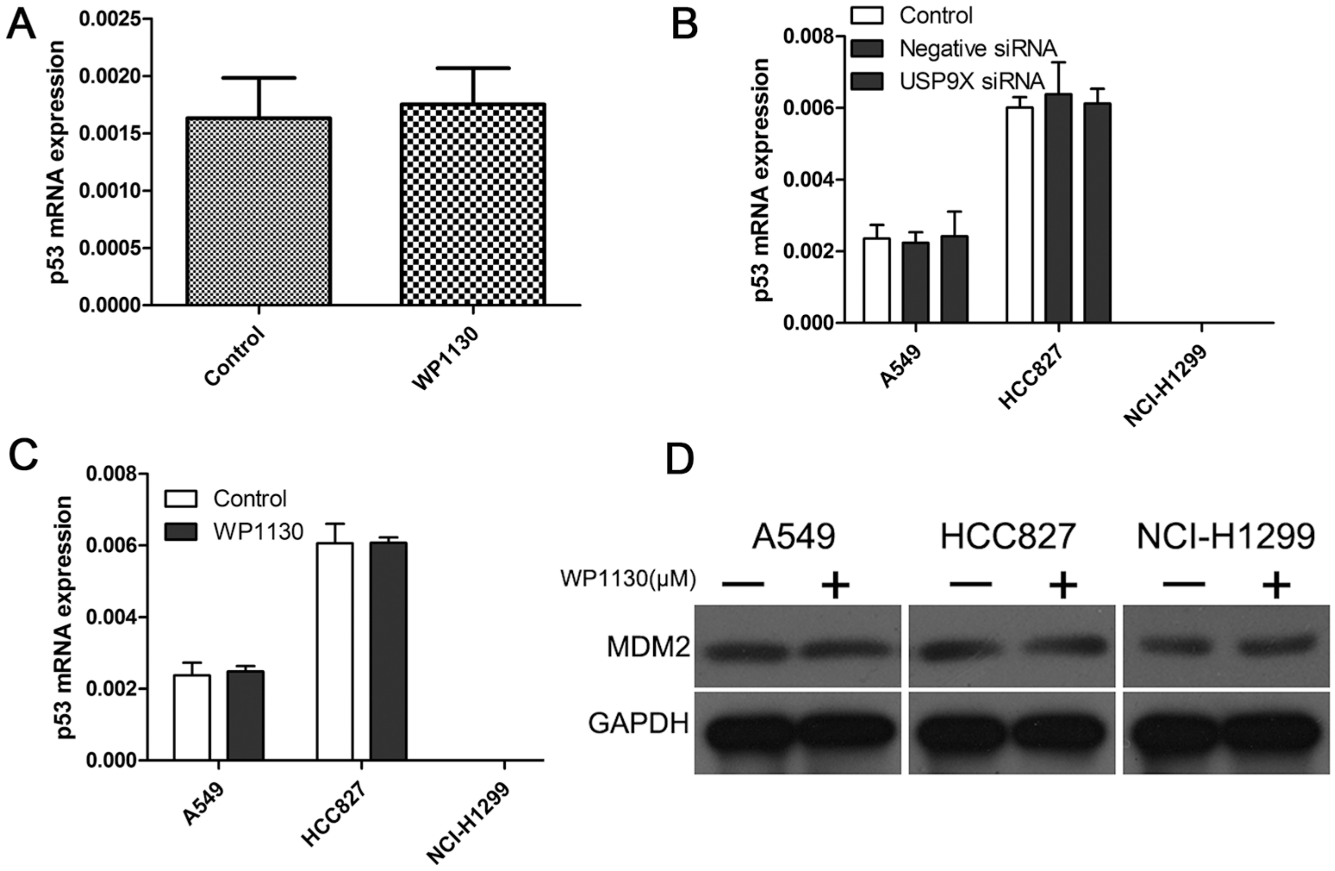

Supplementary Figure 2: (**A**) We detected the expression of p53 mRNA in xenograft tumors through RT-PCR (**B**) RT-qPCR showing p53 mRNA expression in NSCLC cells transfected with USP9X siRNA, a negative control siRNA or control. (**C**) Expression levels of p53 following treatment with or without WP1130 as determined by RT-qPCR. (**D**) Western Blot was used to determine the expression of MDM2 treatment with or without WP1130.
